# Trial by Zoom? The Response to COVID-19 by Canada's Courts

**DOI:** 10.1017/S0008423920000505

**Published:** 2020-05-19

**Authors:** Kate Puddister, Tamara A. Small

**Affiliations:** Department of Political Science, University of Guelph, 50 Stone Rd East, Guelph, Ontario, N1 G 2W1

## Abstract

COVID-19 has made videoconferencing a regular occurrence in the lives of Canadians. Videoconferencing is being used to maintain social ties, run business meetings—and to uphold responsible government. On April 28, 2020, Members of the House of Commons sat virtually using Zoom. The virtual sitting was the first of what will become a stand-in for regular proceedings, allowing the Members to fulfill some of their parliamentary duties while complying with physical distancing (see Malloy, 2020). As the legislative and executive branches look to digital technology to allow the business of government to continue, what about the judicial branch of Canada's government? Courts *are* an essential service. This is best articulated by the Chief Justice of Nova Scotia: “The fact is, the Courts cannot close. As the third branch of government, an independent judiciary is vital for our Canadian democracy to function. It is never more important than in times of crisis” (Wood, 2020). In this analysis, we seek to understand how courts have responded to COVID-19 and the challenges of physical distancing through the use of digital technologies. This is accomplished through a systematic review of COVID-19 statements and directives issued from all levels of court across Canada. We briefly compare Canada to the United States, a jurisdiction that demonstrates greater openness to technology.

## Introduction

COVID-19 has made videoconferencing a regular occurrence in the lives of Canadians. Videoconferencing is being used to maintain social ties, run business meetings—and to uphold responsible government. On April 28, 2020, Members of the House of Commons sat virtually using Zoom. The virtual sitting was the first of what will become a stand-in for regular proceedings, allowing the Members to fulfill some of their parliamentary duties while complying with physical distancing (see Malloy, 2020). As the legislative and executive branches look to digital technology to allow the business of government to continue, what about the judicial branch of Canada's government? Courts *are* an essential service. This is best articulated by the Chief Justice of Nova Scotia: “The fact is, the Courts cannot close. As the third branch of government, an independent judiciary is vital for our Canadian democracy to function. It is never more important than in times of crisis” (Wood, 2020). In this analysis, we seek to understand how courts have responded to COVID-19 and the challenges of physical distancing through the use of digital technologies. This is accomplished through a systematic review of COVID-19 statements and directives issued from all levels of court across Canada. We briefly compare Canada to the United States, a jurisdiction that demonstrates greater openness to technology.

There is a debate in the literature about the extent to which courts should embrace digital technologies. Much of the discussion focuses on the open court principle—that courts must be open (metaphorically and physically) to litigants and the public (Puddister and Small, 2019). Some argue that digital technologies can enhance and change court reporting by allowing journalists to live-tweet or live-blog from the court (Hall-Coates, 2015). Others look to social media to allow courts to create more engaging and transparent relationships with the public (Olsen and O'Clock, 2010). However, concerns have been raised. Former Chief Justice Beverly McLachlin (2012) wondered if court reporting would fall short of journalistic standards of accuracy and fairness if courts allowed the public to use social media to engage in live text-based communication from the courtroom. Others suggest digital technologies and the courts are a clash of cultures, with the former being innovative and the latter being inherently conservative in nature (CCPIO, 2010).

While the circumstances around the use of digital technologies during the pandemic are unique, courts, in Canada and elsewhere, have long reflected on the appropriate use of technology in the justice system. The story of Canadian courts and technology is, at best, a story of slow adaptation, and at worse, one of active resistance. In most Canadian courts, television cameras and live photography are expressly prohibited, and livestreaming of proceedings only regularly occurs in the Supreme Court. This analysis is part of an ongoing effort to understand the Canadian courts in the digital age. In other work, we analyze policies that govern live, text-based communication in courtrooms and the Twitter feeds of Canadian courts (Mattan et al., in press; Puddister and Small, 2019). Our research finds that the Canadian judiciary is very conservative in its approach to digital technology. We find a similar approach during COVID-19.

## Analysis

The judicial system reflects Canada's federal structure. According to the Constitution Act of 1867, provinces have exclusive jurisdiction over lower courts (section 92 courts), the federal government has exclusive jurisdiction over federal courts (section 101 courts), and jurisdiction is shared over superior courts and provincial courts of appeal (section 96 courts). This means each level of court can set policy regarding court administration, which could result in varied approaches to the continuation of service during the COVID crisis, even within a single provincial or territorial jurisdiction. While digital technologies could assist courts in addressing administrative concerns such as the filing of documents, evidence, and scheduling during the pandemic, our analysis focuses only on the adjudicative role or the hearing of legal matters in court. Our comprehensive analysis reviews the policies of all section 101, 96 (trial and appeal), and 92 (trial only) courts, for a total of 44 courts.^1^

Overwhelmingly, we find that Canadian courts have pivoted to provide access to limited essential legal services during the crisis. Almost 91 per cent of courts reviewed are hearing matters deemed “urgent” and “emergency” via technology. While there is some minor variation as to what constitutes urgent and emergency, this commonly covers urgent criminal matters, bail or release from custody, and urgent family matters (Ontario Court of Justice, 2020). Courts are fulfilling their essential service mandate by providing access to matters that are deemed essential, such as the protection of the right to *habeas corpus* (a defendant's right to be brought before a court to justify imprisonment) and the protection of vulnerable individuals within the context of family law. This said, Canadian courts are not necessarily providing “trial by Zoom.” Courts are just as likely to suggest teleconferencing alongside videoconferencing. The decision as to what technology will be used varies. For some courts, a judicial administrator will canvass for the availability of technology among counsel and judges, while others allow the presiding judge to choose. Of note, the Ontario Court of Appeal is using CourtCall, a pay-per-use, third-party service for telephone and video conferencing, and the Nova Scotia Supreme Court recently piloted virtual court using Skype and the court's existing audio system.

Canadian courts are only providing a minimum service during COVID-19. Routine trials, jury trials, and other proceedings have been cancelled or adjourned. The matters that are being heard are largely the jurisdiction of lower trial courts (section 92 and 96 courts); the higher courts of appeal have been slower to adapt, rescheduling matters for summer and fall. For example, the Supreme Court, perhaps Canada's most technologically equipped court, has responded to the COVID-19 challenge by delaying many hearings (Supreme Court of Canada, 2020). Arguably appellate courts should be better suited to transition to online hearings, given the nature of their work, which generally only includes written and oral submissions from counsel (without witnesses or physical evidence). The substantial delay within the legal system that existed prior to COVID-19 will only increase. The case backlog is a serious concern in the family system (Smith, 2020), and in the criminal system, it has been considered a constitutional crisis (see *R. v. Jordan*, 2016). Delay is even more problematic for in-custody individuals due to the institutional transmission of COVID-19, where two-thirds of inmates have tested positive in some institutions (Ivison, 2020). Thus, it is a very real (and potentially deadly) concern that courts cannot continue routine work during the COVID-19 crisis.

The very cautious approach we see in Canada is in marked contrast to the United States. While is it beyond the scope of this analysis to fully discuss what is occurring in the US, American courts are making use of videoconferencing beyond emergency and urgent matters. Some states mandate the use of videoconferencing, while many others are strongly encouraging courts to do so.^2^ For instance, Texas issued an emergency order authorizing courts to conduct proceedings (civil or criminal) through teleconferencing, videoconferencing, or other means, with the exception of jury trials (Texas Judicial Branch, 2020). The emergency order requires that courts ensure public access to court proceedings via technology. Texas judges were provided training on Zoom trials. One possible explanation for the difference in approaches between the two countries is technological; compared to Canada, American courts have been more open to the use of television cameras among other technology (Metz, 1996).

## Discussion

Notwithstanding real concerns regarding delays in the justice system, moving to virtual spaces during COVID-19 is not without its challenges. Indeed, a report produced in collaboration with several American court administration organizations highlights technical and constitutional issues with virtual hearings in the US (Joint Technology Committee, 2020). In terms of technology, privacy issues have been raised regarding the use of Zoom (Joint Technology Committee, 2020). There are also concerns about the quality and reliability of digital technology, which proved to be a concern for Canadian parliamentarians (Malloy, 2020). This raises questions about the digital divide—the inequalities between those who have regular and quality access to digital technologies and those who do not, as well as inequalities in technical competence and skill. Many Canadians, particularly those in rural and remote areas, do not have adequate access to broadband, nor do many Indigenous communities (CRTC, 2020; Hyslop, 2019). These challenges could be potentially insurmountable for an individual attempting to access the court system.

Virtual-only proceedings create particular constitutional challenges. For criminal defendants, the rights to a jury and defence, enshrined in sections 11(f), 7, and 11(d) of the *Charter*, respectively, are undoubtedly limited by online-only proceedings with restrictions placed on legal arguments and evidence, and the complete inability to facilitate a jury trial. Moreover, when proceedings are conducted by telephone or videoconferencing, the ability for the public and the media to participate is limited and open court is restricted. As Paciocco argues, the principle of open court becomes most pressing during times of crisis and when “constitutional rights seem too extravagant to endure” (2005: 387–88), although Texas appears to have found a technological solution to allow for open court.

The COVID-19 crisis has created a considerable challenge for the operation of the judicial system. Across Canada, legal proceedings have been delayed and rescheduled. Most of the work has been at the lower trial courts, which are largely responding only to urgent criminal and family matters. Unlike Parliament, the Supreme Court of Canada has delayed going virtual. However, the COVID challenge presents an opportunity for the court system to innovate and, where appropriate, embrace the capabilities of digital technology. Court administrators might find e-filing and e-scheduling to be more efficient, while correctional and remand institutions might expand video-bail capabilities. As Justice Pringle (Ontario Court of Justice) noted, “One of the silver linings … we feel that we have been booted into the 21st century of technology by this crisis” (in Powell, 2020).

## Notes

1 We reviewed 44 courts: 6 federal courts (section 101); 13 provincial and territorial appellate courts (section 96); 13 provincial and territorial superior trial courts (section 96); and 12 provincial and territorial trial courts (section 92). Note that Nunavut operates a unified trial court (sections 92 and 96 combined).

2 The website of National Center for State Courts features a real-time visual summary of how American state courts are responding to COVID-19 at https://www.ncsc.org/.
